# Assessing the relationship between institutional cancer and diabetes mortality rates using National Death Index data

**DOI:** 10.2144/fsoa-2020-0055

**Published:** 2020-10-20

**Authors:** Nina J Karlin, Matthew R Buras, Heidi E Kosiorek, Kyle E Coppola, Patricia M Verona, Curtiss B Cook

**Affiliations:** 1 Division of Hematology & Medical Oncology, Mayo Clinic Hospital, Phoenix, AZ 85054, USA; 2 Mayo Clinic Cancer Center, Scottsdale, AZ 85259, USA; 3 Biostatistics, Mayo Clinic, Scottsdale, AZ 85259, USA; 4 Department of Information Technology, Mayo Clinic Hospital, Phoenix, AZ 85054, USA; 5 Division of Endocrinology, Mayo Clinic, Scottsdale, AZ 85259, USA

**Keywords:** cancer, endocrinology, outcomes research

## Abstract

**Aim:**

To evaluate overall survival (OS), glycemic control in cancer patients with and without diabetes mellitus (DM).

**Patients & methods:**

Patients (2010–2015) with newly diagnosed prostate, breast, lung, colorectal and pancreatic cancers were identified in institutional cancer registry. Data linked to National Death Index for vital status. 5-year OS estimated; glucose and hemoglobin A_1c_ assessed during year postdiagnosis.

**Results:**

We identified 1404 patients (non-DM, n = 936; DM, n = 468). DM cohort had 168 deaths (36%); non-DM, 267 (29%). 5-year OS estimated at 58% (95% CI: 53–64%) for DM and 67% (95% CI: 64–71%) for controls; for matched pairs, hazard ratio: 1.35 (95% CI: 1.02–1.79). Cancer did not harm glycemic control.

**Conclusion:**

OS among cancer patients with DM was lower than without DM.

## Lay abstract

The aim of this study was to assess how diabetes mellitus (DM) and cancer interact to influence overall survival and glycemic control, through use of a national mortality database, the National Death Index. Institutional records of patients with prostate, lung, breast, colorectal and pancreatic cancers were linked to the National Death Index. Analyses showed that DM was associated with lower overall survival, but a diagnosis of cancer did not worsen glycemic control in patients with or without DM.

**Presented at:** the annual meeting of the Endocrine Society, New Orleans, LA, USA, 23–26 March 2019 (virtual). Portions of this manuscript (abstracts) were published in *Journal of the Endocrine Society* 3(Suppl. 1), April–May 2019, MON-008 (https://doi.org/10.1210/js.2019-MON-008); and 4(Suppl. 1), April–May 2020, MON-116 (https://doi.org/10.1210/jendso/bvaa046.000).

The relationship between diabetes mellitus (DM) and cancer death is unresolved. Examples of inconsistent findings can be found in studies of patients with DM coexisting with colorectal, pancreatic, breast and lung cancers. Some of these studies suggest DM is associated with increased risk of death [1–10]. Differences in study results likely are due to the different methods used to analyze this topic. Some reports have used meta-analyses; others, population- or claims-based data.

Another unresolved question is how cancer affects glycemic control after its diagnosis. Chemotherapeutic regimens, particularly use of glucocorticoids, could lead to acute exacerbations of hyperglycemia in patients with and without DM. By comparison, changes in appetite as a result of chemotherapy may lead to a lowering of blood glucose (BG) levels.

The authors of the present work have previously performed retrospective studies to characterize DM prevalence in their academic-based oncology practice and compared survival among cancer patients with and without DM [11]. Their studies included assessment of individual cancer types (i.e., breast, prostate, lung, pancreatic and colorectal origins), with use of matched case–control methods to compare overall survival (OS) between patients with and without DM. These analyses showed that DM did not significantly impact OS in studies of individual cancer types and no significant effect on BG levels was detected [12–16].

A limitation of these prior assessments was that vital status data were derived from institutional records only. If the patient was lost to follow-up (e.g., moved out of state), the OS estimates may not be accurate. In addition, cause of death was not available from institutional data. To corroborate findings derived from institutional records and determine causes of death, the authors linked their cancer and DM data with the National Death Index (NDI) maintained by the US Centers for Disease Control and Prevention.

## Patients & methods

### Practice overview

The study institution is a National Cancer Institute comprehensive cancer center in Phoenix, Arizona. The full spectrum of cancer care is offered through multidisciplinary collaboration for hematologic malignancies and solid tumors.

### Institutional mortality data acquisition

#### Description of NDI

NDI is a centralized database of death record information on file in state offices of vital statistics [17]. It is a self-supporting service of the National Center for Health Statistics, which is a component of the National Vital Statistics System. NDI assists investigators in the determination of mortality status of persons and provides the cause of death, if applicable. The NDI system selects potential death record matches on the basis of matching criteria and an associated scoring and classification procedure that results in a probabilistic score. Records with scores greater than the recommended cutoff score were considered true matches; records with scores lower than the cutoff scores were considered false matches.

#### Case selection

The Mayo Clinic Institutional Review Board approved this retrospective study. The authors included breast cancer and prostate cancer because these cancers are hormonally mediated tumors. They then expanded the cancer categories to include other common, solid tumor cancers (e.g., colorectal, lung, pancreatic). Cases were truncated in 2015 to allow a sufficient follow-up period for assessment of vital status. Data on prostate, breast, lung, colorectal and pancreatic cancer cases from the calendar year 2010 through 2015 were retrieved from the institutional cancer registry. These cancers were chosen both because of the interest about them and because they were among the most prevalent cancer types in the institutional registry [11].

The institutional registry was established in 1996 to retrospectively collect data elements on all cancer cases. It collects data on age, primary cancer site, histologic characteristics, date of principal cancer diagnosis, sex, race, birthplace and date of death. The registry identifies cases in accordance with pathologic results of malignancy and the *International Classification of Diseases, Ninth Revision* (ICD-9) diagnostic codes. An annual review ensures that information stays up to date.

The present analysis was restricted to death related to prostate, breast, lung, colorectal and pancreatic solid tumors with and without coexisting DM. The relationship between these deaths and DM had already been examined with use of institutional mortality data, which allowed a corroboration of findings with those derived from the NDI [12–16]. However, an expanded data file was generated that included patients from previous analyses plus additional cases. Cancer cases were linked to the electronic health record (EHR) to determine who had a diagnosis of DM using the ICD-9 diagnostic code 250.00, as previously described [12–16]. EHR data were examined around the time of cancer diagnosis to determine who had DM claims during this time period.

Charlson comorbidity index (CCI) was determined for cases and controls on the basis of EHR claims during the ±1 year from the date of cancer diagnosis. The CCI score was modified to exclude DM, to capture the prevalence of comorbid conditions aside from DM in cases and controls [18].

### Statistical analyses

Patients were matched as two controls (cancer with no DM) to one case (cancer with concurrent DM). Patients were matched by diagnosis year, age, sex and cancer type using the Greedy matching algorithm. Eastern Cooperative Oncology Group performance status scores are not available from electronic data and hence were not included. However, with the exception of prostate cancer, previous analyses have shown no statistical differences between DM and non-DM data in performance scores. Additionally, there was little change in DM medications over the year of analysis [12–16]. Therefore, EHRs were not reviewed for these in the present analysis.

Patients with and without DM were compared for demographic characteristics and clinical variables. Continuous variables were compared with paired *t*-tests; categorical variables were compared with McNemar test or Bowker test for symmetry. OS was defined as the time from cancer diagnosis until death from any cause, according to NDI vital statistics. Patients were considered censored at the last known date alive per cancer registry records, if death was not documented per NDI. 5-year OS was estimated with the Kaplan–Meier method. Cox proportional hazards regression model was used to assess the effect of DM on OS and included matched pairs as the strata variables. An additional regression model included a modified CCI score as a covariate. Sample size was based on the number of available cases from 2010 through 2015. It provided 90% power to detect an absolute reduction in survival of 6% or greater at 5 years between cases and controls, across all cancer types combined. Although not statistically powered for individual cancer types, analyses of these data were also undertaken for interest.

To assess changes in glucose control during the year after cancer diagnosis, the authors extracted BG and hemoglobin A_1c_ (HbA_1c_) levels from the laboratory information system. Changes in BG and HbA_1c_ were examined with use of linear mixed models with fixed effects for time, cancer type, DM status and patient-specific random effect, allowing for each patient to have a different intercept. BG was analyzed for patients with and without DM; HbA_1c_ was available only for patients with DM. Statistical software (SAS version 9.4; SAS Institute Inc) was used for analysis. Data were reported as mean standard deviation (SD), median (range) and number (percentage). p < 0.05 was considered statistically significant.

## Results

### Cancer distribution & patient demographic characteristics

**Figure 1.**
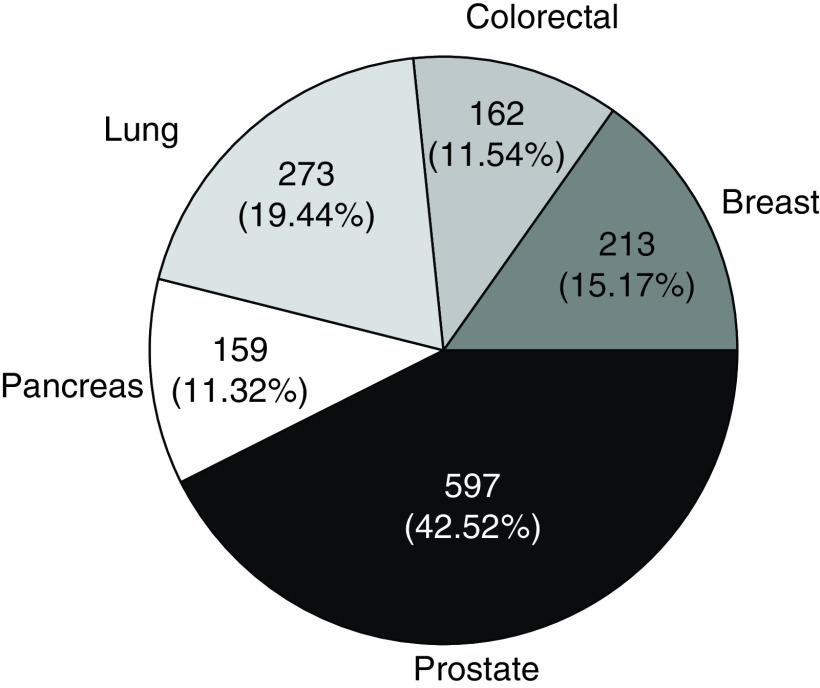
Cancer type distribution (n = 1404). Each cancer type consisted of two to one matching of control patients (n = 936) to patients with diabetes mellitus (n = 468).

Most cases in the dataset were prostate cancer, followed in frequency by lung, breast, colorectal and pancreatic cancers (Figure 1). Mean (SD) age was 68 (10) years for DM and non-DM patients (Table 1). The majority of patients were men (69%) for both cohorts and most patients were white. Cancer stage was similar for both cohorts. CCI without the presence of DM was higher in cases, with a median of 4.0 for cases and 3.0 for controls. Comorbidity prevalence significantly differed between cases and controls for myocardial infarction (13.5 vs 5.7%), congestive heart failure (10.3 vs 5.6%), peripheral vascular disease (19.7 vs 10.3%), chronic pulmonary disease (25.4 vs 18.4%), mild liver disease (18.2 vs 10.8%) and moderate/severe renal disease (13.9 vs 5.0%).

**Table 1.** Patient demographic characteristics overall (n = 1404) and by cancer site.

Characteristic	Diabetes mellitus^†^		p-value
	Yes (n = 468)	No (n = 936)	
Age at diagnosis, mean (SD), years:			
– Overall	67.9 (9.8)	67.8 (9.9)	Matched
Cancer type			
–Breast	66.0 (11.0)	66.0 (10.9)	
– Colorectal	65.3 (9.9)	65.1 (9.7)	
– Lung	71.2 (9.3)	71.2 (9.4)	
– Pancreatic	64.2 (11.8)	63.9 (12.1)	
– Prostate	68.7 (8.3)	68.7 (8.3)	
Sex:			
– Male	328 (70.1)	656 (70.1)	Matched
– Female	140 (29.9)	280 (29.9)	
Race:			
– White	431 (92.1)	879 (93.9)	0.20
– Other than white	37 (7.9)	57 (6.1)	
Ethnicity:			
– Non-Hispanic	438 (93.6)	894 (95.5)	0.12
– Hispanic	30 (6.4)	42 (4.5)	
Stage I/II:			
– Overall	264 (57.1)	576 (62.2)	0.07
– Cancer type			
• Breast	57 (80.3)	121 (85.2)	
• Colorectal	23 (46.0)	44 (43.6)	
• Lung	25 (27.5)	62 (34.3)	
• Pancreatic	23 (43.4)	53 (50.5)	
• Prostate	136 (69.0)	296 (74.6)	
CCI score^‡^, median (range)	3.0 (0–17)	4.0 (0–16)	<0.001

† Values are presented as number (percentage) of patients unless specified otherwise.

‡ CCI modified to exclude presence of diabetes in score.

CCI: Charlson comorbidity index.

### Survival overall & by cancer type

**Figure 2.**
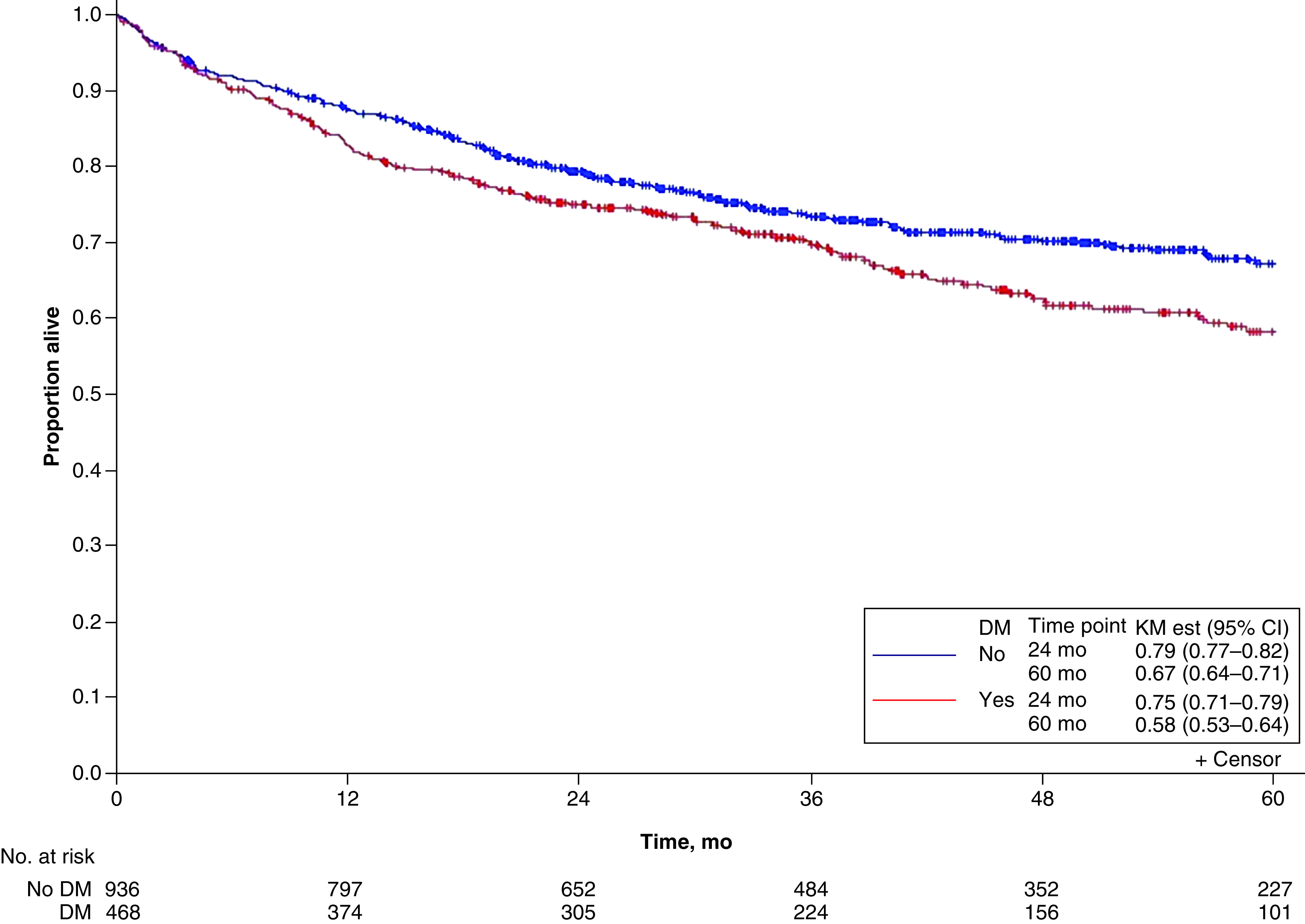
Overall survival for diabetes mellitus. DM: Diabetes mellitus; KM est: Kaplan–Meier estimate.

**Figure 3.**
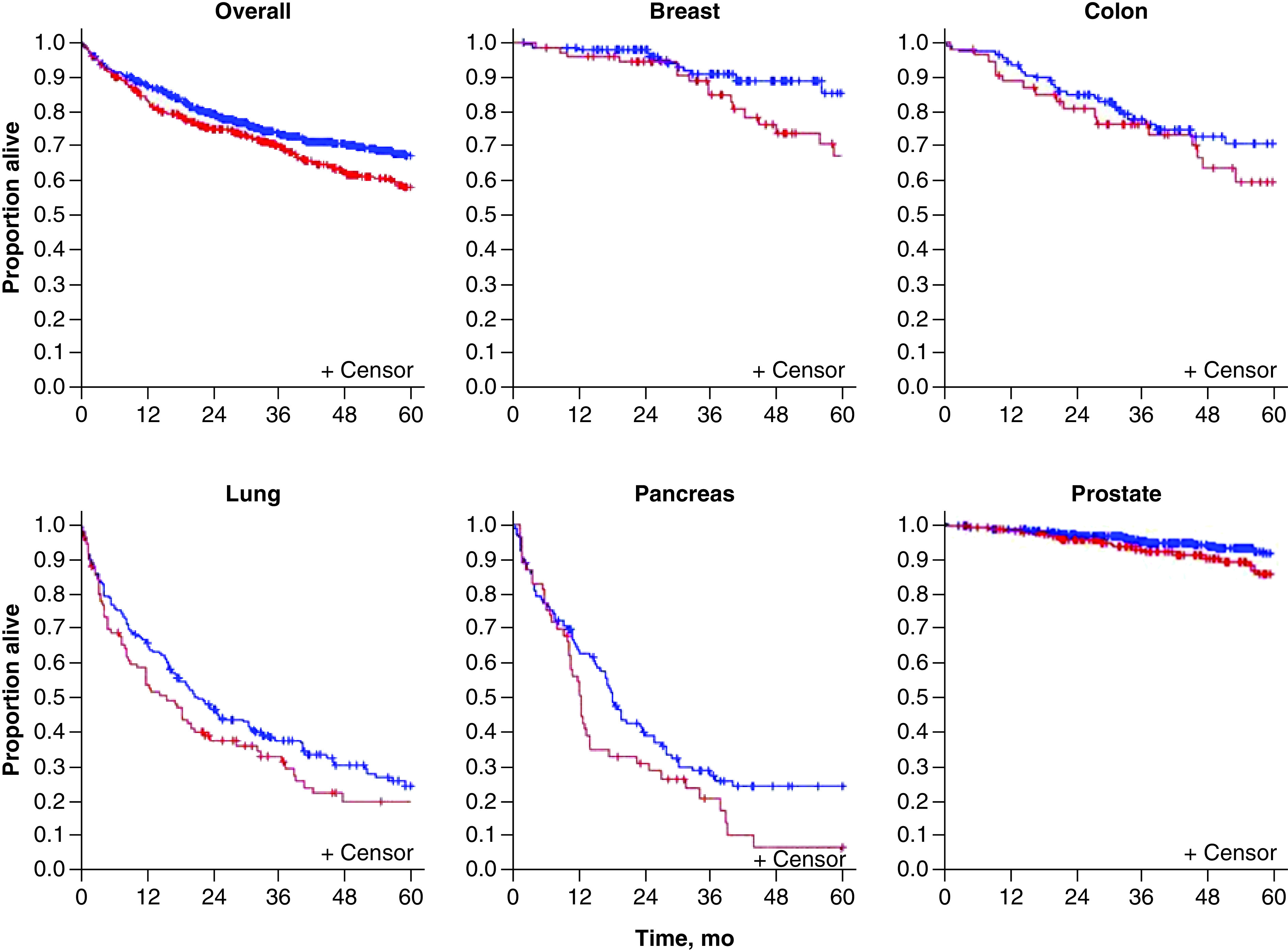
Survival for all cancer types combined and by individual cancer type. The red line denotes patients with diabetes mellitus; the blue line denotes patients who do not have diabetes mellitus.

Overall, 168 deaths occurred among DM patients and 267 among non-DM patients. 5-year OS was estimated at 58% (95% CI: 53–64%) for DM cases and 67% (95% CI: 64–71%) for controls (p = 0.004) (Figure 2). Prostate cancer had the highest 5-year OS at 85% (95% CI: 79–92%) for DM cases and 91% (95% CI: 88–95%) for controls, whereas pancreatic cancer had the lowest at 7% (95% CI: 2–24%) for DM cases and 24% (95% CI: 17–35%) for controls (Figure 3).

**Figure 4.**
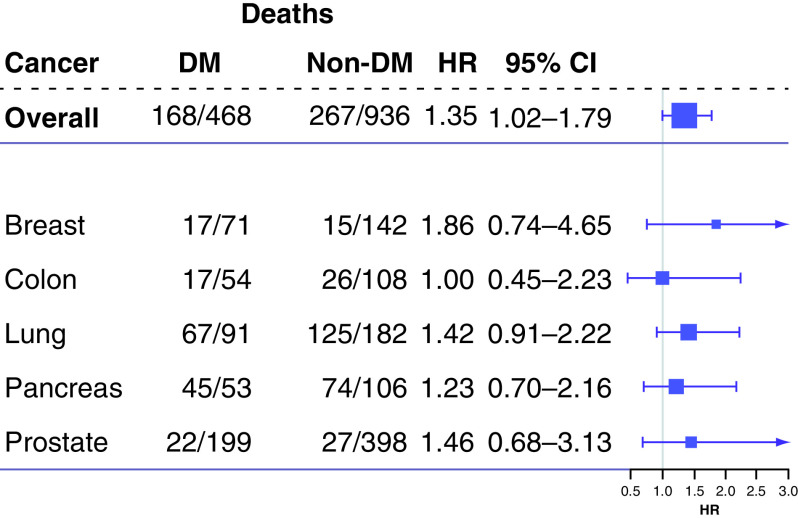
Comparison of survival according to presence of diabetes mellitus. HR is shown as DM versus no DM. DM: Diabetes mellitus; HR Hazard ratio.

Figure 4 provides results on overall death and survival analysis and on death for each cancer type in accordance with presence of DM. Similar to the data shown in Figure 3, a significant difference was observed in OS between DM and non-DM patients when analyzed in aggregate (hazard ratio: 1.35 [95% CI: 1.02–1.79] for matched pairs). When the modified CCI score was included in the model, the hazard ratio for DM was 1.20 (95% CI: 0.88–1.64) with a significant hazard ratio for CCI score of 1.21 (95% CI: 1.13–1.80). For each cancer type, matched-pair Cox regression indicated that the presence of DM did not increase the risk of death for DM patients versus non-DM patients (all p ≥ 0.19).

Overall, cancer-related morbidities were the most common cause of death across all cancer types at 83.9% (Table 2). Vascular-related deaths were the least common deaths for DM patients. Noncancer, nonvascular death was the least common for non-DM patients. Yet, a higher proportion of noncancer, nonvascular deaths occurred among DM patients than non-DM patients. In addition, non-DM patients had a higher proportion of cancer-related deaths and vascular deaths than DM patients.

**Table 2.** Causes of death by diabetes mellitus cohorts.

Cause	Death			p-value
	DM (n = 168)	Non-DM (n = 267)	Total (n = 435)	
Cancer-related	133 (79.2%)	232 (86.9%)	365 (83.9%)	<0.001
Vascular-related	9 (5.4%)	22 (8.2%)	31 (7.1%)	
Noncancer, nonvascular	26 (15.5%)	13 (4.9%)	39 (9.0%)	

DM: Diabetes mellitus.

### Glycemic control

**Figure 5.**
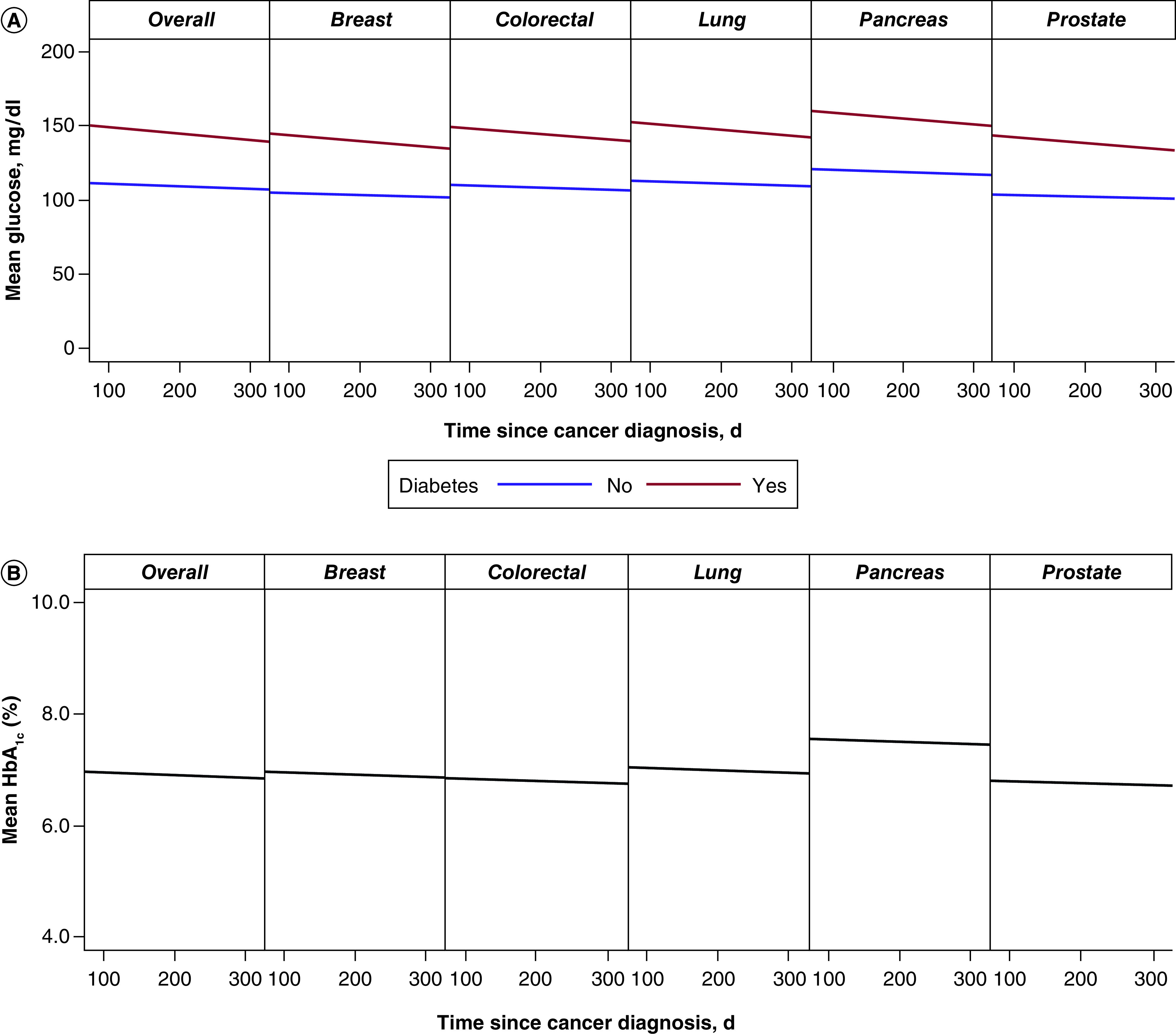
Measurements over first year following diagnosis of various solid organ cancers. **(A)** Blood glucose. Mixed model analysis by site showed days, p < 0.001; diabetes mellitus, p < 0.001; days*diabetes, p = 0.02; site, p < 0.001. Mixed model analysis overall showed days, p < 0.001; diabetes mellitus, p < 0.001; days*diabetes, p = 0.02. **(B)** HbA_1c_. Mixed model analysis by site showed days, p = 0.28; cancer site, p = 0.02. Mixed model analysis overall showed days, p = 0.23. HbA_1c_: Hemoglobin A_1c_.

In the aggregate analysis, mean (SD) BG was 108 (18) mg/dl in the non-DM cohort and 146 (45) mg/dl in the DM cohort. BG decreased over the year for the DM patients and did not change for those without DM (p = 0.02 for interaction effect). Mean (SD) overall HbA_1c_ was 6.9% (1.2%) and no change was observed in HbA_1c_ levels 1 year after cancer diagnosis (p = 0.28) (Figure 5).

Examination of individual cancer types showed that patients with lung and pancreatic cancers had higher BG values overall (p < 0.001) compared with the other types (Figure 5A). A decrease in BG over the year was observed in DM patients for all cancer types (p ≤ .001). Pancreatic cancer patients had higher HbA_1c_ levels overall (p = 0.02) (Figure 5B).

## Discussion

The relationship between DM and death in solid organ cancers has been inconsistent, with some studies showing that DM was associated with decreased survival while others did not show this outcome [1–10]. The literature on solid organ malignancies, DM, death and glycemic control has traditionally treated these characteristics as separate questions analytically. However, in the clinical setting, healthcare teams must consider both diagnoses simultaneously. The authors’ analyses are some of the first to consider both together [12–16]. DM diagnosis did have an impact on OS when all cancer types were considered together, with a 5-year estimated OS of 58% in DM patients and 67% in controls. With control for the impact of modified CCI score, this difference no longer stayed significant, suggestive that the difference seen in OS is based on increasing comorbidity burden associated with DM. The increased association with comorbidities, rather than only the presence of DM, may be what underlies the greater risk of death observed in the data.

Previous analyses have relied on institutionally derived vital status to examine the DM-cancer mortality interaction. The acquisition of these data has been a complex process that required accessing databases from multiple sources. These sources include EHRs, external sources (e.g., family members), state agencies and federal data sources (i.e., Medicare insurance access checks, social security death index checks, external search engines and online obituary checks). The complexity of this process could have led to conflicting data on vital status or incomplete information (e.g., lack of data on cause of death). To overcome potential limitations, the authors used information from a single repository of mortality data – the NDI – that also provided information on cause of death. Despite this analysis using a data selection process slightly different from previous work [12–16], the mortality data and risk conclusions were similar regardless of which source of data – institutional or NDI – was used.

Causes of death of patients with coexisting DM and solid organ cancers have not been explored previously. Although the sample was small, an important cause of death among the DM patients was the noncancer, nonvascular cause. Given that cardiovascular disease is the leading cause of death among patients with DM, the observation that this was not the primary cause of death among the DM patients of the present study might be considered unexpected and requires further investigation [19].

This analysis yielded findings similar to previous reports regarding glycemic control. Among the DM patients, BG was noted to improve over the first year of follow-up and did not change among those without DM. Moreover, HbA_1c_ did not increase in the DM cohort. These findings may have been a result of selection bias. The DM patients had evidence of good glycemic control at baseline. Hence, they may have represented a group already compliant and diligent with their medications and diabetes self-management skills and continued these habits throughout their cancer treatment course. While acute exacerbations of hyperglycemia may occur (e.g., surrounding times of glucocorticoid therapy), patients could be reassured that, at least over the first year of cancer treatment, no significant worsening of BG will occur.

The authors acknowledge some limitations to this study. First, the study was performed retrospectively. Second, determination of whether findings persist over a longer period (e.g., beyond 1 year for glucose control, beyond 5 years for OS) would be important. Third, the results may have limited applicability to other racial and ethnic groups because most patients were white. The DM patients in the cohort represent patients with good glycemic control and results may be different for patients with more severe hyperglycemia. Fourth, it is possible that some non-DM patients had undiagnosed DM or their condition may have been coded incorrectly in the EHR. Fifth, although cases and controls were well matched, the sample sizes for individual cancer types are too small to draw any statistical conclusions and findings should be confirmed in larger cohorts. Sixth, duration of DM, which is associated with greater risk of complications, could have an effect on outcomes. The authors were unable to ascertain the precise timing of DM onset, which typically is self-reported and not accessible in EHRs.

Additionally, differences may exist among the data sources in ascertaining survival status. Although the institutional mortality data may not have tallied some patients who died, the data do gather information from several sources that could corroborate findings. The NDI data, while more expansive, relies on information reported on death certificates, which may not be accurate in reporting DM-related data [20].

Limitations aside, the findings of the present study, based on a national data source, show that DM imposes a greater mortality risk for patients with selected solid organ cancers.

In addition and at least over a short-term follow-up, the presence of malignancy does not significantly worsen glycemic control. These two observations, if confirmed in larger cohort studies, should be an encouraging message for patients and their healthcare teams, in that having DM along with cancer may not adversely affect a patient’s chances of survival.

## Future perspective

The types of analyses described herein need to be extended to a larger, more diverse group of cancers with coexisting DM. In addition, an assessment of other metabolic parameters, such as lipids and blood pressure, could be included. Finally, unknown is whether similar outcomes on death and glycemic control performed with solid organ cancers would be observed for patients with such diagnoses as leukemia and lymphoma.

**Summary points**

- The relationship between diabetes mellitus (DM) and cancer death has conflicting observations.
- Institutional data on patients with breast, lung, prostate, pancreatic and colorectal cancers with and without DM were linked to the National Death Index for vital status and to the institutional laboratory system for glycemic control measures.
- DM was associated with decreased survival across all cancer types.
- Glycemic control did not deteriorate among any of the cancer types.
- Data corroborates earlier studies of individual cancer types regarding death and glycemic control in patients with solid organ cancers and coexisting DM.
